# Porcine hemothropic mycoplasmas infection associated with productive impact in intensive pig production

**DOI:** 10.1186/s40813-020-00171-1

**Published:** 2020-11-04

**Authors:** Fernando Antônio Moreira Petri, Karina Sonalio, Henrique Meiroz de Souza Almeida, Maria Eugênia Silveira Ferraz, Gabriel Yuri Storino, Mauro Rodrigo de Souza, Marcos Rogério André, Luis Guilherme de Oliveira

**Affiliations:** 1 São Paulo State University (Unesp), School of Agricultural and Veterinarian Sciences, Via de Acesso Prof. Paulo Donato Castellane s/n, Jaboticabal, São Paulo 14884-900 Brazil; 2 Evance Animal Health, João Baptista de Queiroz Júnior, 447, Campinas, São Paulo 13098-415 Brazil; 3 Ceva Animal Health, Manoel Joaquim Filho, 303, Paulínia, São Paulo Brazil

**Keywords:** ADWG, Intensive pig production, *Mycoplasma parvum*, *Mycoplasma suis*, qPCR

## Abstract

**Background:**

So far, three porcine hemoplasmas (PH) have been identified, namely *Mycoplasma suis*, *Mycoplasma parvum,* and *Mycoplasma haemosuis.* The first one is the main agent associated with porcine hemoplasmosis, a possible cause of economic losses in pig production. Thus, this work aimed to detect and quantify PH 16S rRNA in finishing pigs and to associate its load estimate with average daily weight gain (ADWG). For this purpose, whole blood samples from 318 pigs were collected at an age of 75 days (d0) when the pigs entered the finishing phase and 105 days later (d105). To calculate ADWG, the animals were weighed at the abovementioned dates. Then, DNA from blood samples were submitted to a qPCR targeting the 16S rRNA gene for PH. Spearman correlation test was performed to investigate potential associations between ADWG and the quantification values. Lastly, the molecular characterization of PH was done by sequencing the 23S rDNA gene.

**Results:**

Out of the 318 samples, 190 (59.74%) were positive on d0, and 304 (95.6%) were positive on d105. A significant correlation was observed (*p* < 0.05), albeit with a low coefficient value (0.18), when comparing ADWG with quantification values on d105. The phylogenetic analysis based on the 23S rDNA gene showed that four sequences were closely related to *M. parvum,* and one sequence was positioned in the *M. suis* cluster.

**Conclusion:**

Two PH, *M. suis* and *M. parvum*, were detected in a Brazilian pig farm. Moreover, increasing occurrence through time was observed, which may have affected the productive performance of positive animals, mainly at the end of the finishing phase, when antimicrobials are removed.

**Supplementary information:**

The online version contains supplementary material available at 10.1186/s40813-020-00171-1.

## Background

Hemotropic mycoplasmas (HMs), also known as hemoplasmas, are commonly associated with infectious anemia in pigs [1]. So far, three hemoplasma species have been described infecting swine, namely *M. suis*, *M. parvum*, and *M. haemosuis* [2–4]. *Mycoplasma suis* is the main agent associated with the swine hemoplasmosis or eperythrozoonosis, which is caused by the pathogen’s adherence to the RBCs surface, triggering the cell’s death [5, 6]. Also, swine hemoplasmosis has been pointed out as a possible cause of economic losses worldwide [1, 6, 7]. On the other hand, *M. parvum* infection has been associated with the absence of clinical signs, even at the peak of bacteremia [8]. Recently, *M. haemosuis* was detected in fattening pigs with skin lesions, fever, and anemia [9]. Even though the pathogenic potential of *M. haemosuis*, the more recently porcine hemoplasma described in China [4], Korea [10], and Germany [9], has not been fully investigated, clinical signs associated with it resemble those previously described for *M. suis* infections [7].

Porcine hemoplasmas (PH) have been described in China [4, 11], the United States [12], Brazil [13–16, 17, 18], Germany [19, 20], France [21], South Korea [10], Japan [22, 23], Hungary [24], Switzerland [25], and Argentina [26]. Additionally, the first three countries, abovementioned, are amongst the four biggest pork producers in the world [27]. In general, PHs are small bacteria found attached to host’s red blood cells, causing, in some cases, deformations to the cell structure and inducing eryptosis [28]. PH-infected swine can present the disease as acute and/or chronic manifestations. While the first is mostly associated with *M. suis* infection and characterized by severe anemia, jaundice, fever, gangrenous ear necrosis, enteritis, hypoglycemia and possible death, the chronic form of the disease seemns to be more common [7]. Moreover, PH infection in pigs may range from asymptomatic to growth retardation, poor reproductive performance, mild anemia, fever, skin lesions, and immunosuppression [3, 9, 27, 28].

Indeed, the chronic presentation of swine hemoplasmosis has been raised as a concern regarding production losses in the finishing phase of pig production, in which the most significant weight gain takes place [29]. Therefore, a reduction in productive and reproductive performance and the predisposition of PH-infected pigs to secondary infections, may lead to financial losses in pig production [4]. Besides, it has been argued that pigs submitted to chronic challenges are prone to present a decrease in feed consumption and feed conversion rates [30]. However, to the best of authors’ knowledge, these effects have not been assessed in hemoplasmas-infected pigs yet.

Even though PHs have been reported in the intensive [18, 31] and extensive [15, 17] pig production systems in Brazil, the influence of these agents on productive swine performance has not been assessed. Therefore, this body of work investigated the occurrence of PH and its association with average daily weight gain (ADWG) in finishing pigs from a commercial farm in Brazil.

## Results

### PH occurrence and load estimate by qPCR

All DNA samples amplified the predicted product for the mammals-*gapdh* gene. Out of the 318 samples, 190 (59.75%) were positive for PHs by the qPCR assay on d0, and 304 (95.60%) were positive on d105. The overall PH incidence was 93.75% (120/128). Among these positive animals, 58.6% were female (75/174) and 35.15% male (45/136) pigs. Besides that, eight (2.52%) animals were negative in both blood sampling, and only six (1.89%) animals were positive on d0 and negative on d105.

Regarding the qPCR assays, all samples were run in duplicate, in 22 different plates, with reaction efficiencies (E) ranging from 92.2 to 102.7%. The analytical assay sensitivity was 10^1^ numbers of PH 16S rRNA copies/μL. The associated slope ranged from − 3.526 to − 3.259, the determination coefficient (*R*^2^) values ranged from 0.987 to 0.998, and the y-Int from 38.58 to 42.1 (Table S1). Cycle quantification values ranged from 19.17 to 39.94 on d0, and the SQ mean values ranged from 1.07 × 10^0^ to 4.30 × 10^6^ PH 16S rRNA copies/μL. Similarly, on d105, Cq values ranged from 15.82 to 39.38, and SQ values ranged from 1.41 × 10^0^ to 4.72 × 10^6^ copies/μL (Table S2). Curiously, a significant difference between SQ values from male and female pigs on d0 and d105 was observed (Table 1).

**Table 1:** Starting quantity values (copies/μL) on d0 and d105 samples

	D0 samples			D105 samples		
	SQ mean	SD	Variance	SQ mean	SD	Variance
Male pigs	5.82 × 10^4a^	±3.99 × 10^5^	1.6 × 10^11^	2.35 × 10^5b^	±1,12 × 10^6^	7.61 × 10^10^
Female pigs	7.21 × 10^3a^	±3.59 × 10^4^	1.3 × 10^9^	8.16 × 10^4b^	±2.76 × 10^5^	1.26 × 10^12^

means followed by different superscript letters indicate significant difference (*p* < 0.05)

**Fig. 1:**
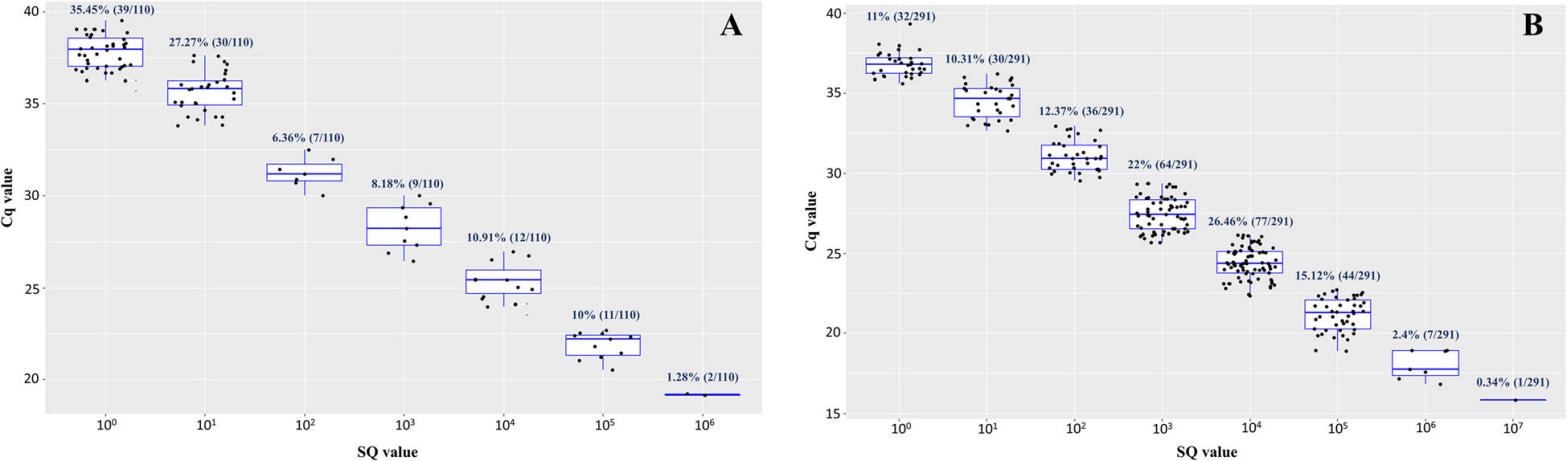
Box plot showing the qPCR Cq results and distribution of SQ values. Quantification cycle (Cq) results and distribution of starting quantity (SQ) degree of dispersion by each sample in qPCR for PH-16S rRNA gene on d0 (**a**) and d105 (**b**)

The samples in which an estimate of PH bacteremia was quantifiable (*n* = 108) were distributed into eight groups (10^0^ to 10^7^), according to SQ values (PH 16S rDNA copies/μL) on d0 and d105 (Fig. 1). It was also noted that 68.5% (74/108) of the positive samples, for which an estimated bacteremia was quantifiable, showed increased SQ values during the finishing phase, whereas 31.48% (34/108) of these samples presented a decrease in the SQ values. Furthermore, 89 (29.28%) out of the 304 positive samples showed inconsistent quantification results even after tested in triplicate (Table S2). This fact is most likely due to the Monte Carlo effect [31], which represents an inherent limitation of the technique, mainly in samples with a low number of porcine PH 16S rDNA copies/μL. Even though PH 16S rDNA could not be quantified in these samples, they were considered positive.

### Associations between PH 16S rDNA quantification and ADWG

**Fig. 2:**
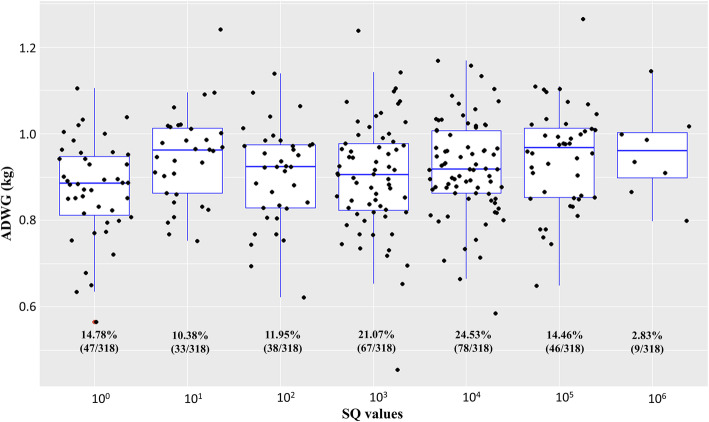
Box plot showing the correlation between ADWG and SQ values on d105. Correlation of starting quantity (SQ) in samples of d105 and distribution of average daily weight gain (ADWG) degree of dispersion by each animal

Regarding the ADWG according to the gender of sampled animals, males showed a mean of 0.95 kg/day (SD = 0.12; Var = 0.015) and females a mean of 0.88 kg/day (SD = 0.12; Var = 0.011). The Wilcoxon test was performed to compare the ADWG and the pigs’ gender, resulting in a significant difference between the means of ADWG of male and female pigs (*p* = 6.24 × 10^− 9^). Besides this, the difference of SQ (d105 – d0) and ADWG mean was significant and positive on the Spearman correlation test (Rho = 0.142; *p* = 0.011) with low coefficient value, indicating that the two variables were weakly correlated. Still, no significant results were observed on the Spearman correlation test between ADWG and SQ mean of blood samples on d0 (*p* = 0.904). However, when comparing ADWG with SQ mean at d105, a significant correlation was observed (*p* = 0.001), but with low coefficient value (0.181) (Fig. 2; Table 2).

**Table 2:** Potential correlations between ADWG and SQ values on d0 and d105

Variables	Coefficient	***P***-value
**SQ**_**1**_ **x SQ**_**2**_	0.08	0.05
**SQ**_**m**_ **x ADWG**	0.14	0.01
**SQ**_**1**_ **x ADWG**	0.007	0.94*
**SQ**_**2**_ **x ADWG**	0.18	0.001

*SQ*_*1*_ Starting quantification value on d0, *SQ*_*2*_ Starting quantification value on d105, *SQ*_*m*_ Starting quantification mean

*There was no correlation

### Molecular characterization of PH

BLASTn analysis for the obtained 23S rDNA sequences showed 91.10 to 99.88% identity with *M. parvum* strain Indiana (NR121958) and 90.10 to 96.54% identity with *M. suis* strain Illinois (NR103970), with query coverage ranging from 98 to 99 (Table 3).

**Table 3:** BLASTn information on the five 23S rDNA sequences obtained from this study

Sample ID	Collection Time	Accession Number	Identity %		Query Cover %
			***M. parvum*** strain Indiana NR121958.1	***M. suis*** strain Illinois NR103970.1	
36	d0	MT 530438	99.65	90.21	99
	d105	MT 530441	99.88	90.39	98
43	d0	MT 530439	91.10	96.54	99
104	d0	MT 530440	99.65	90.18	98
	d105	MT 530442	99.53	90.10	99

**Fig. 3:**
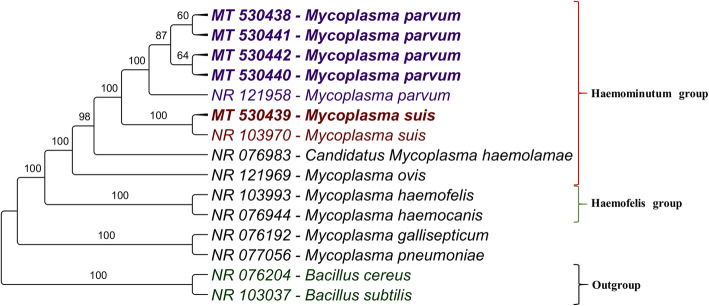
Phylogenetic tree based on *Mycoplasma* spp. 23S rRNA sequences. Phylogenetic analysis based on the Bayesian method, and the TrN + G evolutionary model. Accession numbers are indicated in the sequences. Porcine hemoplasmas sequences detected in the present study are highlighted in bold. Numbers at the nodes correspond to bootstrap

Phylogenetic analysis of the five *Mycoplasma* spp. 23S rDNA sequences, estimated by the Bayesian method, showed that four 23S rDNA sequences were positioned close to *M. parvum* strain Indiana (NR121958) and only one (MT530439) was closely related to *M. suis* strain Illinois (NR103970) (Fig. 3). The numbers at the nodes correspond to bootstrap values accessed with 1.000.000 generations. *Bacillus cereus* and *Bacillus subtilis* were used as outgroup.

## Discussion

Few studies have been conducted regarding the occurrence of PH and its economic impact on commercial pig farms in the world. This is the first report of PH detection by qPCR correlated to the average daily weight gain (ADWG) and its impact on finishing pigs from Brazil.

In the present work, PH was current in 59.75% of the animals at the beginning of the finishing phase, increasing its occurrence through time, reaching 95.60% at the end of this period (d105). Moreover, most animals (68.51%) showed an increase in PH SQ values (copies/μL) from d0 to d105, while 120 animals (37.73%) became positive after d0. These results agreed with previous studies, in which higher occurrence has been observed in older animals [4, 7, 30]. Accordingly, a study conducted in Germany [32] reported a PH occurrence of 31.25% in sows and 14.35% in piglets, suggesting that animals tend to become infected throughout the life. Even though the transmission of PH has been commonly associated with the infestation by the louse *Haematopinus suis* [33], its role in the transmission may have lower importance, since commercial pig farms are very technified and have adequate sanitary measures. As reported in the literature [28, 34], natural infections may be associated with fomites, like the reuse of needles and surgical instruments. Therefore, we believe that mechanical transmission by fomites and direct contact with infected blood, like cannibalism and fights, might have resulted in the transmission of PH between the sampled animals. Indeed, the mechanical transmission of PH may be a problem for the pig production in Brazil, since nearly all finishing animals usually originated from different herds, resulting in the introduction of positive animals and the dissemination of these agents.

Quantitative PCR results showed that more than 80% of the positive animals presented PH loads between 10^− 1^ and 10^4^ copies/μL, corroborating previous studies conducted in Brazil [15, 17, 18, 31]. PHs tend to establish chronic infections with nonclinical presentations in pigs [6, 35]. According to our findings, most animals from this study might have presented a chronic infection characterized by low bacteremia and lack of clinical signs. This information could also explain the high occurrence of PH in Brazil once chronically infected animals play an essential role as a source of the infection. As observed previously, qPCR is very efficient at detecting extremely low bacterial loads and should be used as the standard test for PH detection worldwide. Besides, since conventional PCR and blood smears have demonstrated lower sensitivity and could result in false-negative results, the occurrence of PH in pig farms could be underestimated [12, 20].

Regarding PH infection and its impact on pig production, our results showed that ADWG and bacteremia estimates were weakly correlated, indicating that there might be other pathogens associated with the decrease of ADWG in the evaluated animals. An example could be the respiratory disease caused by *Mycoplasma hyopneumoniae* [36], which has been commonly associated with a decrease in productivity. Furthermore, ADWG of PH-infected male pigs was higher than ADWG from female pigs (0.95 kg/day and 0.88 kg/day, respectively). This could be associated with the body composition, which is quite different between genders, where entire males are more efficient in feed conversion than females [37]. Similarly, when the productive performance of immunologically castrated boars, physically castrated males, entire males, and entire female pigs were compared, the results indicated that immunologically castrated boars were more efficient in feed conversion [38]. Therefore, in this study, the sampled males were previously immune castrated, and so, a higher ADWG was expected compared to the female pigs.

Similarly, to the average daily weight gain, bacteremia estimate levels were higher in male pigs than those found among female pigs in both analyzed phases (75 and 190 days of life). However, we should consider that nearly 90% of the positive animals showed SQ values lower than 10^4^ copies/μL, indicating that these pigs were chronically infected and did not show severe signs of anemia [18]. Besides, it is likely possible that *M. parvum* is the primary pathogen associated with PH in Brazil, and this PH does not cause any clinical signs, even at the peak of the bacteremia, and yet, seems to persist at low levels in the blood [8]. On the other hand, when comparing ADWG with SQ mean on d105, a significant correlation was observed. This fact may be associated with the withdrawal of antimicrobials 15 days before slaughter, aiming at avoiding the presence of residues in the meat. Besides, antimicrobials as metaphylaxis may also control PH infection and its productive impact on the finishing phase, allowing a higher multiplication only after its complete withdrawal. Considering that antimicrobial use in pig production is a big concern, many countries have prohibited its use as growth promoters and disease prevention. Therefore, it is possible that diseases inhibited by antimicrobials’ preventive use may emerge and become a serious problem in pig production worldwide.

The phylogenetic tree based on the 23S rDNA gene of hemoplasmas showed the occurrence of both *M. suis* and *M. parvum* in the studied herd. The concomitant presence of both PH has been previously reported in sows from southern Brazil [30]. When comparing *M. suis* strain Illinois to *M. parvum* strain Indiana, bacteremia levels, at the peak, were one log lower for *M. parvum* than *M. suis* [8]*.* Additionally, these two pathogens are genetically related, and the genome of *M. parvum* has orthologous for all the protein-coding sequences (CDS) with metabolic functions identified in the genome of *M. suis* [8]. Still, a set of unique paralogous CDS of *M. suis* have a higher percentage of signal peptides detected than *M. parvum* CDS, which could play an important role in the pathogenicity of the first [8, 12]. Considering the high degree of genetic similarity between *M. suis* and *M. parvum*, it is crucial to use less conserved gene fragments, e.g. 23S rDNA, to assess the phylogenetic positioning of these agents, and thus, get a better picture of PH occurrence in swine herds worldwide.

## Conclusion

Porcine hemoplasmas occurrence increased through the finishing phase, showing an inverse correlation between PH 16S rDNA quantification values at d105 and average daily weight gain. Besides, the occurrence of two porcine hemoplasmas in the same herd, namely *M. suis* and *M. parvum*, was reported, indicating possible coinfection.

## Methods

### Study design and sampling

The study was carried out from November 2018 through March 2019 in a pig production farm belonging to an integration system, located in Patos de Minas (18.5873°S, 46.5147°W), Minas Gerais State, southeastern Brazil. It was conducted with the approval of the School of Agricultural and Veterinarian Sciences’ Animals Ethics Committee (CEUA) under permit number 073778/19. Appropriate permission was obtained from the farm owners before collection of blood samples from the animals.

In total, 318 pigs (male and female) from the same batch were selected at the beginning of the rearing/finishing phase, approximately 75 days of age (d0). The selected pigs were identified with ear tags with random numbering and weighed at the beginning and at the end of the finishing phase (d105), to determine the average daily weight gain (ADWG). The animals were slaughtered at 180 days of life (d105), and for each animal included in the assay, a whole blood sample was collected in Vacutest® tubes, containing ethylenediaminetetraacetic acid (EDTA). Blood collection was carried out on two occasions: first, at the beginning of the finishing phase (d0) and, second, at the end of this phase, in a slaughterhouse (d105). The animals have no obvious clinical symptoms during blood sampling. After sampling, the blood samples were aliquoted in cryogenic tubes, conditioned in liquid nitrogen and transported to the Swine Medicine Laboratory, in the Department of Veterinary Clinic of the Faculty of Agricultural and Veterinary Sciences (FCAV– UNESP), *campus* of Jaboticabal, where they were stored at − 80 °C until processing.

### DNA extraction and conventional (c) PCR for the mammals-gapdh gene

DNA extraction was performed as previously described protocol [39]. To rule out the presence of inhibitors in the extracted DNA samples and the occurrence of false negative in the qPCR for PHs, all samples were submitted to a conventional PCR (cPCR) using the *gapdh*-F primer oligonucleotides (5′-CCTTCATTGACCTCAACTACAT-3′) and *gapdh*-R (5′-CCAAAGTTGTCATGGATGACC3’), which flank a fragment of the mammals-*gapdh* gene [40]. The amplified products were subjected to horizontal electrophoresis on 1% agarose gels stained with Ethidium Bromide (1 μL/20 mL) (Life Technologies™, Carlsbad, California, USA) in TBE pH 8.0 running buffer at a current of 90 V/50 mA for 50 min. Then, the amplified products were visualized using an ultraviolet light transilluminator (Chemi-Doc, Bio-Rad®).

### Porcine hemoplasmas-qPCR based on the 16S rRNA gene

DNA samples positive in the cPCR for the endogenous mammals-*gadph* gene were subjected to a qPCR assay based on the 16S rDNA coding gene for PH, using the primer oligonucleotides (Integrated DNA Technologies®, Coralville, Iowa, USA) F (5′- CCCTGATTGTACTAATTGAATAAG-3′) and R (5′-GCGAACACTTGT-TAAGCAAG-3′) and the TaqMan hydrolysis probe (5’FAM- TGRATACACAYTTCAG-MGBNFQ3’ [12]. The amplification reaction was performed according to the protocol described in the literature [12], completed in a CFX 96 Thermocycler (BioRad®).

Ten-fold serial dilutions were performed, from 10^7^ copies/μL until 10^1^ copies/μL, to determine the different concentrations of IDT SMART plasmids (Integrated DNA Technologies, Coralville, Iowa, USA) containing the target sequence. In all qPCR assays, plasmids and sterile ultrapure water were used as positive and negative control, respectively (Nuclease-Free Water, Promega®, Madison, United States).

### cPCR for *Mycoplasma* spp. based on the 23S rRNA gene

To amplify a 800pb fragment of *Mycoplasma* spp. 23S rRNA gene fragment, a cPCR assay, was performed using the primers 23S_HAEMO_F (5′- TGAGGGAAAGAGCCCAGAC-3′) and 23S_HAEMO_R (5-’-GGACAGAATTTACCTGACAAGG-3′), described by Mongruel et al. (unpublished). The amplification reaction contained 1x PCR buffer, 1.5 mM of MgCl_2_, 0.2 mM of each dNTP, 0.4 mM of each primer, 2.5 U of Taq Platinum DNA Polymerase (Life Technologies™, California, USA), 5 μL of DNA template, and ultra-pure water q.s.p 25 μL. The cycling conditions consisted of 3 min denaturation at 94 °C followed by 35 cycles of 94 °C for 30 s, 54 °C for 30 s and 72 °C for 60 s, with a final extension of 72 °C for 10 min. The amplified product was visualized in 1% agarose gel as above described on the cPCR for the *gapdh* gene section.

### Sequencing and sequence analyses

Amplified products were purified using the “Exosap IT” kit (Applied Biosystems, Cleveland, Ohio, USA) according to the manufacturer’s recommendations. The sequencing of amplified products was performed according to the method described in existent literature [41]. The resulting sequences were then submitted to a screening test using Phred-Phrap software version 23 [42, 43] to check for the chromatogram quality. BLAST program [44] was used to analyze the nucleotides’ sequence and to search for the percentage of identity with previously deposited sequences in GenBank [45].

### Phylogenetic analyses

The 23S rRNA sequences originated from this study, and those retrieved from GenBank were aligned using MAFFT software [46]. The Bayesian inference (BI) analysis was performed with MrBayes 3·1·2 [47] via CIPRES Science Gateway [48]. The best evolutionary model was selected by the program jModelTest2 (version 2.1.6) on XSEDE [49], under the Akaike Information Criterion (AIC) [50]. The tree was edited in TreeGraph 2.0 β [51]. The bootstrapping values were indicated at the nodes, based on 1.000.000 generations. The number of generations was selected based on the value of the average standard deviation of split frequencies (between 0.01 and 0.05) according to MrBayes version 3.2 Manual [47].

### Data analysis

To detect potential correlations between PH prevalence and continuous variables, data normality was assessed using the Shapiro-Wilkins test (*p* < 0.05). While the Pearson correlation coefficient (*p* < 0.05) was used to detect significant correlations if the data presented normal distribution, Spearman’s rank coefficient test (*p* < 0.05) was used for non-parametric data. To compare the ADWG and the pigs’ gender, the Wilcoxon test was performed. The abovementioned analysis was performed using the software R version 3.5.1 [52]. Moreover, to find the PH incidence value, the number of new cases between d0 and d105 was divided by the number of negative animals from the first blood sampling.

## Supplementary information

Additional file 1: **Table S1.** Parameters of qPCR assays based on 16S rRNA gene from pigs on d0 and d105. **Table S2.** Detailed information on qPCR results of the 318 samples at d0 and d105. (DOCX 112 kb)

## Data Availability

The *M. suis* and *M. parvum* partial genome sequence obtained in this study had been uploaded to GenBank. The datasets used and/or analysed during the current study are available from the corresponding author on reasonable request.

## Abbreviations

16S rDNA: 16S ribosomal DNA
16S rRNA: 16S ribosomal RNA
ADWG: Average daily weight gain
CDS: Protein-coding sequences
cPCR: Conventional polymerase chain reaction
Cq: Quantification cycle
DNA: Deoxyribonucleic acid
EDTA: Ethylenediaminetetraacetic acid
HM: Hemothropic mycoplasma
*M. haemosuis*: *Mycoplasma haemosuis*
*M. parvum*: *Mycoplasma parvum*
*M. suis*: *Mycoplasma suis*
PH: Porcine hemoplasmas
qPCR: Quantitative real time polymerase chain reaction
SQ: Starting quantification
TBE: Tris-borate-EDTA
