# Supplementary material for: Porcine hemothropic mycoplasmas infection associated with productive impact in intensive pig production
Source: Porcine Health Manag. 2020 Nov 4;6:33. doi: 10.1186/s40813-020-00171-1 (PMC7641829; doi:10.1186/s40813-020-00171-1)
Supplement: Supplementary file 1 — Table S1. Parameters of qPCR assays based on 16S rRNA gene from pigs on d0 and d105. Table S2. Detailed information on qPCR results of the 318 samples at d0 and d105. (DOCX 112 kb) [file 40813_2020_171_MOESM1_ESM.docx]

**Supplementary information**

**Table S1:** Parameters of qPCR assays based on 16S rRNA gene from pigs on d0 and d105.

| **Plate** | **E (%)** | **R²** | **Slope** | **y-inter** |
| --- | --- | --- | --- | --- |
| 1 | 96.5 | 0.996 | -3.410 | 40.93 |
| 2 | 97.3 | 0.997 | -3.388 | 39.86 |
| 3 | 102.7 | 0.995 | -3.259 | 40.13 |
| 4 | 97.0 | 0.987 | -3.395 | 40.52 |
| 5 | 98.7 | 0.996 | -3.353 | 40.06 |
| 6 | 96.0 | 0.995 | -3.422 | 41.58 |
| 7 | 93.0 | 0.989 | -3.502 | 42.10 |
| 8 | 92.2 | 0.997 | -3.526 | 42.00 |
| 9 | 101.2 | 0.995 | -3.293 | 41.03 |
| 10 | 97.7 | 0.996 | -3.378 | 39.89 |
| 11 | 98.1 | 0.996 | -3.369 | 39.57 |
| 12 | 94.4 | 0.992 | -3.463 | 39.74 |
| 13 | 98.2 | 0.994 | -3.366 | 39.59 |
| 14 | 100.2 | 0.997 | -3.318 | 39.61 |
| 15 | 98.1 | 0.988 | -3.367 | 39.06 |
| 16 | 97.6 | 0.992 | -3.380 | 39.41 |
| 17 | 96.5 | 0.996 | -3.410 | 39.83 |
| 18 | 98.5 | 0.995 | -3.359 | 39.23 |
| 19 | 100.2 | 0.998 | -3.318 | 38.58 |
| 20 | 93.9 | 0.988 | -3.477 | 39.94 |
| 21 | 99.9 | 0.994 | -3.324 | 39.14 |
| 22 | 99.3 | 0.996 | -3.339 | 38.98 |

r^2^: determination coefficient. E (%): efficiency

**Table S2**: Detailed information on qPCR results of the 318 samples at d0 and d105.

| **Sample ID** | **75 days of age (d0)** | |  | **180 days of age (d105)** | |  |  |  |  |
| --- | --- | --- | --- | --- | --- | --- | --- | --- | --- |
|  | **Cq mean** | **SQ mean** |  | **Cq mean** | **SQ mean** |  | **Sex** |  | **ADWG (kg)** |
| 2 | - | - |  | 34,67 | 2,86E+01 |  | **M** |  | 0,979 |
| 3 | - | - |  | 24,12 | 3,20E+04 |  | **M** |  | 1,018 |
| 4 | 35,92 | 1,25E+01 |  | 25,39 | 1,37E+04 |  | **M** |  | 0,919 |
| 5 | 36,30 | 1,13E+01 |  | 22,84 | 7,51E+04 |  | **M** |  | 0,902 |
| 6 | 34,64 | 3,50E+01 |  | 16,79 | 4,26E+06 |  | **M** |  | 0,934 |
| 7 | 36,93 | 6,96E+00 |  | 27,17 | 5,06E+03 |  | **M** |  | 0,917 |
| 8 | 37,14 | 1,53E+01 |  | 25,09 | 2,08E+04 |  | **M** |  | 1,070 |
| 10* | 35,77 | 1,62E+01 |  | 29,32 | 1,15E+03 |  | **M** |  | 0,454 |
| 11* | 35,85 | 1,53E+01 |  | 23,99 | 4,34E+04 |  | **M** |  | 0,876 |
| 12 | 34,30 | 3,66E+01 |  | 19,84 | 7,50E+05 |  | **M** |  | 1,006 |
| 13 | - | - |  | 21,33 | 2,72E+05 |  | **M** |  | 0,930 |
| 14 | - | - |  | 35,26 | 1,95E+01 |  | **M** |  | 0,960 |
| 15 | 38,00 | 3,70E+00 |  | 22,47 | 1,25E+05 |  | **M** |  | 1,046 |
| 16* | 35,18 | 2,41E+01 |  | 33,80 | 5,43E+01 |  | **M** |  | 0,965 |
| 17 | 35,83 | 3,82E+01 |  | 15,82 | 1,16E+07 |  | **M** |  | 0,999 |
| 18 | - | - |  | 30,33 | 5,72E+02 |  | **M** |  | 0,995 |
| 19 | 33,84 | 6,04E+01 |  | 22,43 | 1,26E+05 |  | **M** |  | 1,010 |
| 20 | 30,02 | 8,04E+02 |  | 25,58 | 1,46E+04 |  | **M** |  | 1,013 |
| 21 | - | - |  | 33,06 | 8,80E+01 |  | **M** |  | 1,241 |
| 23 | 38,19 | 4,65E+00 |  | 23,84 | 4,81E+04 |  | **M** |  | 0,918 |
| 24 | 23,95 | 3,59E+04 |  | 24,24 | 3,75E+04 |  | **M** |  | 1,076 |
| 26 | 36,67 | 7,57E+00 |  | 27,69 | 3,45E+03 |  | **M** |  | 0,898 |
| 27* | 34,70 | 3,35E+01 |  | 27,39 | 4,23E+03 |  | **M** |  | 1,048 |
| 29 | 37,11 | 6,50E+00 |  | 33,30 | 5,74E+01 |  | **M** |  | 1,022 |
| 30 | - | - |  | 26,10 | 1,04E+04 |  | **M** |  | 0,892 |
| 31 | 25,45 | 1,80E+04 |  | 27,34 | 4,50E+03 |  | **M** |  | 0,925 |
| 32 | 28,22 | 2,73E+03 |  | 19,83 | 7,52E+05 |  | **M** |  | 1,096 |
| 33* | 38,94 | 4,34E+00 |  | 31,11 | 3,38E+02 |  | **M** |  | 0,972 |
| 34* | 38,90 | 4,46E+00 |  | 32,68 | 1,14E+02 |  | **M** |  | 1,139 |
| 35 | - | - |  | 30,10 | 6,62E+02 |  | **M** |  | 1,095 |
| 36 | 19,23 | 4,30E+06 |  | 17,13 | 4,72E+06 |  | **M** |  | 1,144 |
| 37 | - | - |  | 26,33 | 8,73E+03 |  | **M** |  | 0,695 |
| 38 | - | - |  | 34,89 | 2,55E+01 |  | **M** |  | 1,018 |
| 40 | 37,08 | 7,56E+00 |  | 30,01 | 7,05E+02 |  | **M** |  | 1,064 |
| 41* | 37,36 | 5,46E+00 |  | 21,75 | 1,79E+05 |  | **M** |  | 1,033 |
| 42 | 38,13 | 2,99E+00 |  | 22,36 | 1,35E+05 |  | **M** |  | 1,103 |
| 43 | 21,03 | 3,62E+05 |  | 21,23 | 2,92E+05 |  | **M** |  | 1,266 |
| 44 | - | - |  | 23,33 | 6,89E+04 |  | **M** |  | 0,797 |
| 45 | - | - |  | 20,78 | 3,91E+05 |  | **M** |  | 1,010 |
| 49 | 38,61 | 5,46E+00 |  | 27,39 | 4,23E+03 |  | **M** |  | 1,142 |
| 50* | 38,24 | 6,19E+00 |  | 36,84 | 6,71E+00 |  | **M** |  | 1,000 |
| 51* | 38,13 | 6,65E+00 |  | 24,14 | 3,93E+04 |  | **M** |  | 0,850 |
| 52 | 35,09 | 5,19E+01 |  | 34,34 | 3,94E+01 |  | **M** |  | 1,002 |
| 53 | - | - |  | 26,06 | 1,22E+04 |  | **M** |  | 1,034 |
| 55* | 39,14 | 3,75E+00 |  | 22,54 | 1,42E+05 |  | **M** |  | 0,849 |
| 56 | - | - |  | 29,12 | 1,46E+03 |  | **M** |  | 0,964 |
| 58 | - | - |  | 34,22 | 3,13E+01 |  | **M** |  | 1,091 |
| 59 | 38,14 | 8,18E+00 |  | 32,04 | 1,95E+02 |  | **M** |  | 0,768 |
| 60* | 39,29 | 3,04E+00 |  | 24,38 | 3,99E+04 |  | **M** |  | 0,871 |
| 61 | 37,16 | 8,83E+00 |  | 32,48 | 1,41E+02 |  | **M** |  | 1,012 |
| 62 | - | - |  | 33,94 | 3,72E+01 |  | **M** |  | 0,985 |
| 63 | - | - |  | 26,78 | 7,64E+03 |  | **M** |  | 0,907 |
| 64* | 39,94 | 1,96E+00 |  | 25,97 | 1,30E+04 |  | **M** |  | 1,169 |
| 65 | 37,27 | 1,20E+01 |  | 21,66 | 2,58E+05 |  | **M** |  | 0,959 |
| 66 | 34,30 | 8,83E+01 |  | 30,03 | 7,77E+02 |  | **M** |  | 0,955 |
| 67 | - | - |  | 25,00 | 2,60E+04 |  | **M** |  | 0,954 |
| 68 | - | - |  | 24,95 | 2,63E+04 |  | **M** |  | 0,877 |
| 69 | - | - |  | 20,52 | 5,66E+05 |  | **M** |  | 1,073 |
| 70* | 38,63 | 4,76E+00 |  | 28,70 | 1,39E+03 |  | **M** |  | 1,042 |
| 71* | 37,47 | 1,04E+01 |  | 36,18 | 1,09E+01 |  | **M** |  | 0,938 |
| 72 | 39,03 | 4,39E+00 |  | 22,24 | 1,74E+05 |  | **M** |  | 0,853 |
| 75* | 39,19 | 3,26E+00 |  | 30,59 | 5,37E+02 |  | **M** |  | 0,914 |
| 76 | - | - |  | 35,94 | 1,30E+01 |  | **M** |  | 1,016 |
| 78 | 37,29 | 1,23E+01 |  | 21,38 | 3,20E+05 |  | **M** |  | 1,104 |
| 79 | - | - |  | 21,37 | 3,15E+05 |  | **M** |  | 0,944 |
| 80* | 39,66 | 2,37E+00 |  | 32,92 | 1,05E+02 |  | **M** |  | 0,973 |
| 81 | - | - |  | 30,22 | 6,92E+02 |  | **M** |  | 1,040 |
| 83* | 39,10 | 3,46E+00 |  | 27,16 | 5,66E+03 |  | **M** |  | 0,896 |
| 85 | - | - |  | 26,15 | 1,15E+04 |  | **M** |  | 0,917 |
| 87* | 38,33 | 5,80E+00 |  | 27,93 | 3,33E+03 |  | **M** |  | 0,833 |
| 88 | 25,44 | 1,92E+04 |  | 23,21 | 8,77E+04 |  | **M** |  | 1,134 |
| 89 | 38,76 | 4,42E+00 |  | 24,77 | 3,04E+04 |  | **M** |  | 1,007 |
| 90* | 39,54 | 1,28E+00 |  | 25,71 | 1,56E+04 |  | **M** |  | 0,997 |
| 91 | 38,96 | 1,96E+00 |  | 26,51 | 8,99E+03 |  | **M** |  | 1,074 |
| 92* | 38,58 | 4,91E+00 |  | 25,03 | 2,51E+04 |  | **M** |  | 0,890 |
| 93 | 22,33 | 2,92E+05 |  | 25,06 | 2,44E+04 |  | **M** |  | 0,952 |
| 94 | - | - |  | 23,71 | 6,24E+04 |  | **M** |  | 1,009 |
| 95 | 29,33 | 2,06E+03 |  | - | 3,47E+03 |  | **M** |  | 1,238 |
| 96 | - | - |  | 28,40 | 2,40E+03 |  | **M** |  | 0,887 |
| 97 | 38,25 | 3,82E+00 |  | 19,99 | 8,26E+05 |  | **M** |  | 0,851 |
| 98 | 38,04 | 4,44E+00 |  | 22,08 | 1,11E+05 |  | **M** |  | 0,999 |
| 99* | 38,48 | 3,22E+00 |  | 30,91 | 2,65E+02 |  | **M** |  | 0,976 |
| 100 | 37,01 | 7,18E+00 |  | 26,80 | 4,46E+03 |  | **M** |  | 1,027 |
| 102* | 38,53 | 2,53E+00 |  | 24,25 | 2,50E+04 |  | **M** |  | 0,811 |
| 103 | 27,55 | 7,26E+03 |  | 36,53 | 5,68E+00 |  | **M** |  | 0,753 |
| 104 | 21,22 | 3,39E+05 |  | 17,73 | 2,81E+06 |  | **M** |  | 0,910 |
| 106 | 36,04 | 1,43E+01 |  | 30,23 | 4,24E+02 |  | **M** |  | 0,985 |
| 107 | 21,46 | 5,57E+05 |  | 25,18 | 1,37E+04 |  | **M** |  | 0,895 |
| 108 | - | - |  | 23,10 | 5,54E+04 |  | **M** |  | 0,875 |
| 109 | 37,68 | 5,83E+00 |  | 25,10 | 1,42E+04 |  | **M** |  | 1,032 |
| 110 | - | - |  | 26,09 | 7,23E+03 |  | **M** |  | 0,875 |
| 111 | 38,29 | 3,69E+00 |  | 21,01 | 2,32E+05 |  | **M** |  | 0,977 |
| 112 | - | - |  | 27,85 | 2,14E+03 |  | **M** |  | 0,908 |
| 116 | - | - |  | 29,98 | 5,10E+02 |  | **M** |  | 0,923 |
| 117* | 37,07 | 8,74E+00 |  | 18,89 | 9,81E+05 |  | **M** |  | 1,110 |
| 118 | - | - |  | 34,69 | 2,04E+01 |  | **M** |  | 0,831 |
| 119 | 37,42 | 6,91E+00 |  | 21,04 | 2,26E+05 |  | **M** |  | 0,779 |
| 120 | 30,70 | 8,03E+02 |  | 31,15 | 2,26E+02 |  | **M** |  | 0,881 |
| 121* | 37,23 | 7,76E+00 |  | 36,01 | 8,97E+00 |  | **M** |  | 0,774 |
| 122 | 36,04 | 1,80E+01 |  | 23,09 | 6,76E+04 |  | **M** |  | 1,104 |
| 123* | 38,45 | 2,68E+00 |  | 18,87 | 9,95E+05 |  | **M** |  | 0,992 |
| 124 | 39,04 | 1,87E+00 |  | 28,49 | 1,39E+03 |  | **M** |  | 0,947 |
| 126 | 22,40 | 2,80E+05 |  | 32,29 | 1,18E+02 |  | **M** |  | 0,972 |
| 128 | 35,00 | 3,78E+01 |  | 23,37 | 4,58E+04 |  | **M** |  | 1,158 |
| 129 | 36,64 | 1,19E+01 |  | 25,67 | 9,72E+03 |  | **M** |  | 0,769 |
| 130 | - | - |  | 24,01 | 2,97E+04 |  | **M** |  | 1,019 |
| 131* | 38,56 | 3,04E+00 |  | 37,10 | 4,12E+00 |  | **M** |  | 0,901 |
| 132 | - | - |  | 30,33 | 3,93E+02 |  | **M** |  | 0,925 |
| 133* | 38,29 | 3,69E+00 |  | 23,96 | 3,06E+04 |  | **M** |  | 0,927 |
| 134 | - | - |  | 31,84 | 1,40E+02 |  | **M** |  | 0,622 |
| 135 | - | - |  | 27,59 | 2,56E+03 |  | **M** |  | 0,856 |
| 136* | 38,56 | 3,04E+00 |  | 24,20 | 2,60E+04 |  | **M** |  | 0,918 |
| 138* | 38,57 | 3,02E+00 |  | 24,05 | 2,92E+04 |  | **M** |  | 0,895 |
| 140 | 37,03 | 7,10E+00 |  | 23,96 | 3,07E+04 |  | **M** |  | 0,755 |
| 142 | 37,90 | 4,21E+00 |  | 26,04 | 8,74E+03 |  | **M** |  | 1,069 |
| 143 | 19,17 | 2,02E+06 |  | 25,46 | 1,10E+04 |  | **M** |  | 1,088 |
| 144 | 36,81 | 1,29E+01 |  | 22,41 | 8,95E+04 |  | **M** |  | 0,946 |
| 147* | 37,40 | 8,29E+00 |  | 27,30 | 3,85E+03 |  | **M** |  | 0,935 |
| 148 | 36,19 | 1,25E+01 |  | 22,32 | 1,15E+05 |  | **M** |  | 1,022 |
| 150* | 39,13 | 2,57E+00 |  | 26,35 | 7,39E+03 |  | **M** |  | 1,074 |
| 151* | 35,27 | 3,51E+01 |  | 28,48 | 1,76E+03 |  | **M** |  | 0,653 |
| 155 | - | - |  | 26,11 | 8,36E+03 |  | **M** |  | 0,814 |
| 160* | 37,97 | 5,63E+00 |  | 23,76 | 4,38E+04 |  | **M** |  | 0,959 |
| 162 | 29,57 | 1,72E+03 |  | 31,73 | 1,90E+02 |  | **M** |  | 0,924 |
| 165* | 31,45 | 4,68E+02 |  | 24,37 | 2,88E+04 |  | **M** |  | 0,665 |
| 167 | - | - |  | 22,12 | 1,31E+05 |  | **M** |  | 1,023 |
| 168* | 36,81 | 1,24E+01 |  | 28,32 | 1,96E+03 |  | **M** |  | 0,952 |
| 171* | 37,41 | 8,26E+00 |  | 24,81 | 2,11E+04 |  | **M** |  | 0,969 |
| 176 | 26,52 | 1,33E+04 |  | 36,47 | 7,49E+00 |  | **M** |  | 0,816 |
| 177 | - | - |  | - | - |  | **M** |  | 0,779 |
| 181 | - | - |  | 21,35 | 2,23E+05 |  | **M** |  | 0,989 |
| 183 | - | - |  | 22,92 | 7,64E+04 |  | **M** |  | 0,929 |
| 184 | - | - |  | 35,13 | 1,87E+01 |  | **M** |  | 0,910 |
| 186* | - | - |  | 37,31 | 4,21E+00 |  | **M** |  | 0,985 |
| 190 | 31,16 | 5,70E+02 |  | 21,91 | 1,56E+05 |  | **M** |  | 0,976 |
| 192 | - | - |  | 25,33 | 1,49E+04 |  | **M** |  | 1,056 |
| 199 | 35,60 | 2,93E+01 |  | 26,27 | 7,78E+03 |  | **M** |  | 0,766 |
| 201* | 39,09 | 2,63E+00 |  | 20,85 | 3,13E+05 |  | **F** |  | 0,996 |
| 202* | 37,75 | 6,56E+00 |  | 25,54 | 1,28E+04 |  | **F** |  | 0,863 |
| 203* | 37,00 | 1,09E+01 |  | 24,38 | 2,82E+04 |  | **F** |  | 0,879 |
| 204 | 24,39 | 5,69E+04 |  | 23,27 | 6,07E+04 |  | **F** |  | 0,931 |
| 208 | 27,34 | 7,60E+03 |  | 26,23 | 7,99E+03 |  | **F** |  | 0,965 |
| 210* | 35,34 | 3,35E+01 |  | 20,17 | 4,96E+05 |  | **F** |  | 0,850 |
| 211 | 39,04 | 2,82E+00 |  | 23,14 | 6,70E+04 |  | **F** |  | 0,948 |
| 214 | 37,62 | 7,16E+00 |  | 23,71 | 4,53E+04 |  | **F** |  | 0,968 |
| 215 | - | - |  | 27,36 | 3,76E+03 |  | **F** |  | 1,097 |
| 216 |  |  |  | 25,76 | 1,13E+04 |  | **F** |  | 1,024 |
| 220 |  |  |  | 33,44 | 5,88E+01 |  | **F** |  | 0,986 |
| 222 | 37,34 | 5,72E+00 |  | 33,28 | 6,56E+01 |  | **F** |  | 1,095 |
| 224 | - | - |  | 36,22 | 7,61E+00 |  | **F** |  | 0,963 |
| 226* | 36,26 | 1,36E+01 |  | 26,18 | 8,24E+03 |  | **F** |  | 0,981 |
| 227 | - | - |  | 22,18 | 1,27E+05 |  | **F** |  | 1,069 |
| 230 |  |  |  | 25,76 | 1,06E+04 |  | **F** |  | 1,031 |
| 231 |  |  |  | 28,73 | 1,80E+03 |  | **F** |  | 0,959 |
| 232 |  |  |  | 22,62 | 1,12E+05 |  | **F** |  | 0,954 |
| 234 |  |  |  | 36,19 | 7,75E+00 |  | **F** |  | 0,942 |
| 235 |  |  |  | 18,88 | 1,40E+06 |  | **F** |  | 0,987 |
| 236* | 38,01 | 3,61E+00 |  | 27,75 | 3,57E+03 |  | **F** |  | 0,853 |
| 238 | 26,91 | 8,64E+03 |  | 29,74 | 9,10E+02 |  | **F** |  | 0,807 |
| 240 | - | - |  | 35,97 | 1,41E+01 |  | **F** |  | 0,946 |
| 242 | 35,78 | 1,65E+01 |  | 21,70 | 2,09E+05 |  | **F** |  | 0,833 |
| 243 | 35,09 | 3,04E+01 |  | 35,61 | 1,15E+01 |  | **F** |  | 0,903 |
| 246 |  |  |  | 20,74 | 4,09E+05 |  | **F** |  | 0,922 |
| 247 | 34,30 | 4,52E+01 |  | 20,28 | 5,43E+05 |  | **F** |  | 0,856 |
| 248 | - | - |  | 31,29 | 3,21E+02 |  | **F** |  | 0,985 |
| 249 | - | - |  | 23,11 | 8,11E+04 |  | **F** |  | 1,022 |
| 301* | 37,27 | 6,79E+00 |  | 22,72 | 1,05E+05 |  | **F** |  | 0,910 |
| 302 | - | - |  | 23,94 | 4,61E+04 |  | **F** |  | 0,960 |
| 303 | - | - |  | 24,32 | 3,56E+04 |  | **F** |  | 1,007 |
| 304* | - | - |  | 37,40 | 5,18E+00 |  | **F** |  | 0,942 |
| 305 | - | - |  | 28,38 | 2,30E+03 |  | **F** |  | 0,846 |
| 306 | - | - |  | 25,80 | 1,31E+04 |  | **F** |  | 0,800 |
| 307* | 37,06 | 7,83E+00 |  | 26,55 | 8,16E+03 |  | **F** |  | 0,873 |
| 308* | 37,87 | 4,49E+00 |  | 21,74 | 2,04E+05 |  | **F** |  | 0,954 |
| 309 | 24,91 | 3,33E+04 |  | 26,50 | 8,18E+03 |  | **F** |  | 0,990 |
| 311 | 22,55 | 1,38E+05 |  | 26,12 | 1,05E+04 |  | **F** |  | 0,916 |
| 312* | 24,54 | 4,27E+04 |  | 39,16 | 1,58E+00 |  | **F** |  | 0,929 |
| 313 | - | - |  | 28,16 | 2,65E+03 |  | **F** |  | 0,905 |
| 314 | 24,51 | 3,60E+04 |  | 36,40 | 5,97E+00 |  | **F** |  | 0,679 |
| 315 | 22,68 | 1,53E+05 |  | 24,83 | 2,51E+04 |  | **F** |  | 0,967 |
| 316 | - | - |  | 26,76 | 6,88E+03 |  | **F** |  | 0,768 |
| 317 | - | - |  | 20,24 | 5,59E+05 |  | **F** |  | 0,780 |
| 318 | 30,01 | 1,01E+03 |  | 27,29 | 4,81E+03 |  | **F** |  | 0,917 |
| 319* | 36,70 | 1,00E+01 |  | 37,39 | 5,24E+00 |  | **F** |  | 0,894 |
| 320* | 37,17 | 7,26E+00 |  | - | - |  | **F** |  | 0,647 |
| 321 | 37,61 | 1,45E+01 |  | 39,33 | 1,41E+00 |  | **F** |  | 0,952 |
| 322 | - | - |  | 33,35 | 4,91E+01 |  | **F** |  | 1,019 |
| 324 | - | - |  | 28,16 | 2,73E+03 |  | **F** |  | 0,718 |
| 325 | - | - |  | 25,94 | 8,07E+03 |  | **F** |  | 1,105 |
| 326* | 38,49 | 8,04E+00 |  | 37,33 | 3,69E+00 |  | **F** |  | 0,957 |
| 327 | - | - |  | 31,66 | 1,82E+02 |  | **F** |  | 0,841 |
| 328 | - | - |  | 33,01 | 6,13E+01 |  | **F** |  | 0,934 |
| 329 | - | - |  | 30,63 | 3,66E+02 |  | **F** |  | 0,923 |
| 330* | - | - |  | 36,15 | 8,31E+00 |  | **F** |  | 0,795 |
| 331* | - | - |  | 37,21 | 4,01E+00 |  | **F** |  | 0,722 |
| 332 | 38,50 | 7,98E+00 |  | - | - |  | **F** |  | 0,814 |
| 333 | 37,57 | 1,49E+01 |  | 26,34 | 6,97E+03 |  | **F** |  | 0,818 |
| 334 | 36,94 | 7,51E+00 |  | 31,05 | 2,74E+02 |  | **F** |  | 0,834 |
| 335 | - | - |  | 25,16 | 1,38E+04 |  | **F** |  | 1,043 |
| 336 | 22,49 | 1,43E+05 |  | 27,13 | 4,10E+03 |  | **F** |  | 0,837 |
| 337* | 37,85 | 1,24E+01 |  | 39,38 | 9,08E-01 |  | **F** |  | 0,841 |
| 338* | 39,46 | 4,19E+00 |  | 37,01 | 4,72E+00 |  | **F** |  | 0,887 |
| 339 | - | - |  | 37,13 | 4,24E+00 |  | **F** |  | 0,799 |
| 340 | 26,96 | 1,90E+04 |  | 24,34 | 2,43E+04 |  | **F** |  | 0,902 |
| 342 | - | - |  | 37,71 | 2,93E+00 |  | **F** |  | 0,771 |
| 343 | 31,45 | 3,17E+02 |  | 31,85 | 1,60E+02 |  | **F** |  | 0,950 |
| 344 | 28,84 | 5,43E+03 |  | 34,32 | 2,95E+01 |  | **F** |  | 0,908 |
| 345* | 38,31 | 9,08E+00 |  | 24,53 | 2,40E+04 |  | **F** |  | 0,850 |
| 346 | - | - |  | 36,80 | 4,68E+00 |  | **F** |  | 0,882 |
| 347 | - | - |  | 30,92 | 3,01E+02 |  | **F** |  | 0,694 |
| 348 | - | - |  | - | - |  | **F** |  | 1,020 |
| 349 | 26,74 | 2,24E+04 |  | 29,14 | 1,02E+03 |  | **F** |  | 0,730 |
| 350 | - | - |  | 26,32 | 7,07E+03 |  | **F** |  | 0,944 |
| 352 | - | - |  | - | - |  | **F** |  | 0,891 |
| 354 | - | - |  | 37,15 | 3,57E+00 |  | **F** |  | 1,032 |
| 355 | - | - |  | 37,51 | 2,79E+00 |  | **F** |  | 0,851 |
| 356 | - | - |  | - | - |  | **F** |  | 0,958 |
| 359* | 38,83 | 6,38E+00 |  | 37,39 | 3,66E+00 |  | **F** |  | 1,038 |
| 360 | - | - |  | 17,57 | 2,59E+06 |  | **F** |  | 1,017 |
| 364 | - | - |  | 30,13 | 5,17E+02 |  | **F** |  | 0,866 |
| 367 | - | - |  | 36,92 | 4,58E+00 |  | **F** |  | 0,885 |
| 368 | - | - |  | 33,92 | 3,29E+01 |  | **F** |  | 0,824 |
| 369* | - | - |  | 38,28 | 1,93E+00 |  | **F** |  | 0,635 |
| 370 | - | - |  | 36,14 | 8,43E+00 |  | **F** |  | 1,020 |
| 371 | - | - |  | - | - |  | **F** |  | 0,914 |
| 372 | - | - |  | 19,70 | 6,60E+05 |  | **F** |  | 0,978 |
| 375* | 39,01 | 7,61E+00 |  | 35,34 | 1,49E+01 |  | **F** |  | 0,807 |
| 376* | 38,18 | 2,59E+00 |  | - | - |  | **F** |  | 0,882 |
| 378 | - | - |  | 24,57 | 1,70E+04 |  | **F** |  | 0,840 |
| 379* | - | - |  | 38,08 | 1,42E+00 |  | **F** |  | 0,856 |
| 381 | - | - |  | 22,35 | 7,84E+04 |  | **F** |  | 0,817 |
| 383* | 37,78 | 1,72E+01 |  | 36,73 | 3,70E+00 |  | **F** |  | 0,871 |
| 387 | - | - |  | 35,03 | 1,52E+01 |  | **F** |  | 0,752 |
| 388* | 38,56 | 1,03E+01 |  | 35,87 | 8,59E+00 |  | **F** |  | 0,887 |
| 389 | - | - |  | - | - |  | **F** |  | 0,838 |
| 390 | - | - |  | 37,70 | 2,44E+00 |  | **F** |  | 0,850 |
| 391* | 37,46 | 2,12E+01 |  | 38,32 | 1,21E+00 |  | **F** |  | 0,823 |
| 392 | 36,84 | 6,53E+00 |  | - | - |  | **F** |  | 0,834 |
| 394 |  |  |  | 35,30 | 1,27E+01 |  | **F** |  | 0,863 |
| 395 | - | - |  | 36,03 | 5,93E+00 |  | **F** |  | 0,930 |
| 398* | - | - |  | 38,95 | 7,79E-01 |  | **F** |  | 0,769 |
| 399 | 38,85 | 1,86E+00 |  | - | - |  | **F** |  | 0,792 |
| 400* | 39,33 | 6,16E+00 |  | 36,06 | 7,67E+00 |  | **F** |  | 0,955 |
| 429 | - | - |  | 25,31 | 1,24E+04 |  | **F** |  | 0,962 |
| 519 | 33,81 | 5,14E+01 |  | 23,91 | 2,72E+04 |  | **F** |  | 0,714 |
| 607 | - | - |  | 33,00 | 4,84E+01 |  | **F** |  | 0,969 |
| 611 | - | - |  | - | - |  | **M** |  | 0,935 |
| 619 | - | - |  | 29,51 | 5,48E+02 |  | **F** |  | 0,964 |
| 621 | - | - |  | 35,18 | 1,09E+01 |  | **F** |  | 1,011 |
| 628 | 31,98 | 7,79E+02 |  | 26,13 | 5,70E+03 |  | **F** |  | 1,015 |
| 631 | - | - |  | 25,02 | 1,23E+04 |  | **F** |  | 0,930 |
| 640* | 36,55 | 3,83E+01 |  | 35,92 | 6,39E+00 |  | **F** |  | 0,851 |
| 644* | 39,29 | 1,22E+00 |  | 35,16 | 1,41E+01 |  | **F** |  | 1,061 |
| 647* | 39,25 | 6,52E+00 |  | 20,59 | 2,75E+05 |  | **F** |  | 0,903 |
| 830 | 34,92 | 2,43E+01 |  | 26,17 | 5,53E+03 |  | **M** |  | 1,000 |
| 863* | 39,10 | 1,39E+00 |  | 19,58 | 5,37E+05 |  | **F** |  | 0,975 |
| 865* | 39,16 | 6,92E+00 |  | 35,60 | 8,02E+00 |  | **F** |  | 1,004 |
| 874 | - | - |  | 23,26 | 4,17E+04 |  | **F** |  | 0,885 |
| 876 | 22,22 | 1,42E+05 |  | 24,44 | 1,87E+04 |  | **M** |  | 0,890 |
| 882 | - | - |  | 23,97 | 2,54E+04 |  | **M** |  | 0,819 |
| 883 | - | - |  | 31,12 | 1,78E+02 |  | **M** |  | 0,744 |
| 887 | - | - |  | 36,27 | 5,09E+00 |  | **F** |  | 0,650 |
| 888 | - | - |  | 28,38 | 1,22E+03 |  | **M** |  | 0,969 |
| 890* | 35,78 | 1,71E+01 |  | 27,47 | 2,25E+03 |  | **M** |  | 0,861 |
| 891* | 37,14 | 6,82E+00 |  | 32,65 | 7,88E+01 |  | **F** |  | 0,963 |
| 900 | 21,79 | 1,91E+05 |  | 25,68 | 7,75E+03 |  | **F** |  | 0,972 |
| 904 | 26,44 | 9,14E+03 |  | 27,12 | 4,91E+03 |  | **F** |  | 0,735 |
| 905* | 38,64 | 2,50E+00 |  | 27,92 | 2,89E+03 |  | **F** |  | 1,028 |
| 911 | 36,89 | 6,30E+00 |  | 23,18 | 6,81E+04 |  | **F** |  | 0,809 |
| 912 | 36,26 | 9,62E+00 |  | 28,11 | 2,63E+03 |  | **F** |  | 0,829 |
| 913 | 25,04 | 2,06E+04 |  | 24,40 | 2,99E+04 |  | **F** |  | 0,707 |
| 915* | 38,91 | 1,57E+00 |  | 19,91 | 5,86E+05 |  | **F** |  | 0,761 |
| 916* | 35,59 | 1,93E+01 |  | 29,92 | 7,76E+02 |  | **F** |  | 0,937 |
| 918 | - | - |  | 24,11 | 3,61E+04 |  | **F** |  | 0,898 |
| 920* | 38,65 | 2,48E+00 |  | 28,51 | 1,95E+03 |  | **F** |  | 0,746 |
| 923 | 30,89 | 3,81E+02 |  | 29,33 | 1,13E+03 |  | **F** |  | 0,848 |
| 924 | - | - |  | 29,13 | 1,29E+03 |  | **F** |  | 0,923 |
| 925* | 37,42 | 5,68E+00 |  | 24,35 | 3,09E+04 |  | **F** |  | 0,790 |
| 926 | - | - |  | 27,73 | 3,33E+03 |  | **F** |  | 0,810 |
| 927 | 34,15 | 5,10E+01 |  | 29,35 | 1,11E+03 |  | **F** |  | 0,882 |
| 928 | - | - |  | 30,60 | 4,90E+02 |  | **F** |  | 0,885 |
| 929 | - | - |  | 32,79 | 1,15E+02 |  | **F** |  | 0,827 |
| 932 | 39,53 | 1,07E+00 |  | - | - |  | **F** |  | 0,865 |
| 934 | - | - |  | 21,06 | 2,71E+05 |  | **F** |  | 0,649 |
| 936 | - | - |  | 22,37 | 1,14E+05 |  | **F** |  | 0,866 |
| 938 | 35,05 | 2,31E+01 |  | 21,71 | 1,77E+05 |  | **F** |  | 0,832 |
| 939* | 36,27 | 1,22E+01 |  | 25,06 | 1,93E+04 |  | **F** |  | 0,733 |
| 946 | 39,04 | 1,95E+00 |  | 25,30 | 1,66E+04 |  | **F** |  | 0,586 |
| 947 | - | - |  | 20,16 | 4,92E+05 |  | **F** |  | 0,810 |
| 949 | 36,74 | 8,92E+00 |  | 30,47 | 3,55E+02 |  | **F** |  | 0,768 |
| 951 | - | - |  | 27,93 | 2,91E+03 |  | **F** |  | 0,825 |
| 953* | 37,24 | 4,92E+00 |  | 29,31 | 1,17E+03 |  | **F** |  | 0,977 |
| 954 | 32,48 | 1,57E+02 |  | 36,26 | 6,75E+00 |  | **F** |  | 0,894 |
| 957 | - | - |  | 36,87 | 7,67E+00 |  | **F** |  | 0,891 |
| 958 | 35,88 | 1,26E+01 |  | 32,74 | 1,20E+02 |  | **F** |  | 0,829 |
| 959* | 39,35 | 1,55E+00 |  | 26,67 | 6,58E+03 |  | **F** |  | 0,790 |
| 961* | 38,88 | 1,61E+00 |  | 20,54 | 3,34E+05 |  | **F** |  | 0,745 |
| 962 | 36,65 | 9,54E+00 |  | 38,04 | 1,91E+00 |  | **F** |  | 0,565 |
| 964 | - | - |  | 23,01 | 7,46E+04 |  | **F** |  | 0,960 |
| 965 | - | - |  | - | - |  | **F** |  | 0,888 |
| 969* | 39,51 | 1,05E+00 |  | 18,91 | 1,13E+06 |  | **F** |  | 0,866 |
| 976* | 37,32 | 4,66E+00 |  | 24,32 | 3,15E+04 |  | **F** |  | 0,863 |
| 984* | 36,26 | 9,96E+00 |  | 38,10 | 1,83E+00 |  | **F** |  | 0,832 |
| 985* | 38,07 | 2,98E+00 |  | 23,48 | 5,50E+04 |  | **F** |  | 0,876 |
| 989 | 37,65 | 4,06E+00 |  | 35,52 | 1,89E+01 |  | **F** |  | 0,795 |
| 990 | 25,43 | 1,35E+04 |  | 25,69 | 1,13E+04 |  | **F** |  | 0,862 |
| 993 | - | - |  | 30,93 | 3,05E+02 |  | **F** |  | 0,805 |
| 1051* | 36,64 | 7,73E+00 |  | 37,76 | 2,60E+00 |  | **F** |  | 0,808 |
| 1052 | - | - |  | 24,24 | 3,28E+04 |  | **F** |  | 0,828 |
| 1057 | 38,76 | 4,96E+00 |  | 37,95 | 3,42E+00 |  | **F** |  | 0,870 |
| 1062* | 36,46 | 8,69E+00 |  | 27,86 | 2,53E+03 |  | **F** |  | 0,998 |
| 1065 | 35,93 | 3,64E+01 |  | 28,49 | 1,67E+03 |  | **F** |  | 0,818 |
| 1071 | 20,52 | 3,51E+05 |  | 35,80 | 1,06E+01 |  | **F** |  | 0,860 |
| 1073 | 37,99 | 4,46E+00 |  | 30,92 | 2,98E+02 |  | **F** |  | 0,753 |
| 1085 | 35,27 | 1,95E+01 |  | 22,81 | 8,44E+04 |  | **F** |  | 0,845 |
| 1090* | 37,53 | 1,16E+01 |  | 36,48 | 6,32E+00 |  | **F** |  | 1,105 |
| 1099 | - | - |  | 26,52 | 6,27E+03 |  | **F** |  | 0,799 |
| 1100 | - | - |  | 18,92 | 1,22E+06 |  | **F** |  | 0,798 |

*These samples presented the Monte Carlo Effect.
